# Peninsular Journal and Dr. Storer

**Published:** 1856-09

**Authors:** 


*Peninsular Journal and Dr. Storer.*—In the Peninsular Journal of Medicine for June, 1856, page 564, appear the following editorial remarks:

"*Humphreys R. Storer, Professor of Midwifery in the M. M. College, Boston.*—It is so rarely that one sees admitted into the columns of the National Intelligencer, even in the way of advertisement, anything disorganizing or pernicious in its tendencies, that my attention was specially arrested by the appearance of a card in that venerable journal, giving notice to the inhabitants of Washington, of the arrival in that city of a German Homœopathist, by the name of Perdbeau, recently from Boston. But the surprise naturally excited by the insertion of the notice in an organ uniformly so conservative, was soon merged in a feeling of disgust on learning by its perusal that this Teutonic Quack was permitted to refer to Humphreys R. Storer, of Boston. We say permitted, for we can hardly conceive that even Homœopathic impudence, famous everywhere for its unblushing effrontery, would dare perpetrate such an act of audacity as to refer, without his consent, to a gentleman whose name was to be used, and the influence of whose public professional position was to be invoked in aid of the designs of the impostor.

"This statement is made as a reason for instituting the inquiry, what relation this Dr. Storer bears to the medical profession of Boston? And whether it is possible that one of this name affiliated to our frend, H. R. Storer, M.D., can have so far forgotten the respect due his natural and professional allegiance, as to allow his name to be merged in the advertisement of a mountebank?

"Boston can answer for itself, but for us, who live in the neighborhood of the granaries of the West, we have learned to separate the wheat from the tares, and have also learned one other thing from the customs of rural life, that of keeping a partition wall between the wolves and the flock."

The contemptuous and insulting tone of the above paragraphs would deserve no notice, were it not for certain facts which we will specify.

In the first place, Dr. Zina Pitcher, the senior editor of the Peninsular Journal, is too well acquainted with the professional character of Dr. Storer to believe, for a moment, anything derogatory to the honorable bearing and keen conscientiousness of feeling which uniformly characterize his course, and have done so through the whole of his medical career. Had he been a stranger to him, there might be some excuse for so offensive an attack; but, even then, the gentlemanly and. courteous mode of procedure would have been a private note of inquiry to the supposed offender. *En passant*, we may say that the astonishing perversity in misspelling and misnaming which characterize the effusion above quoted, and others in this and several Western journals, is a little ludicrous, and quite unnecessary. The editors of the Peninsular knew very well, could they only have put off a certain slip-shod habit of arranging their sentences—which, by the way, is aptly copied by their compositors and proof-readers—that Professor Storer's name is not what they have made it; and, moreover, the name of "*Perdbeau*" is, we have his authority for saying, wholly unknown to him. We have the charity, however, to believe that it is a careless reading and printing for Per*a*beau.

We are content to concur with the graceful and courteous writer of the editorial note quoted, in his remark as to the capability of Boston to answer for itself, and sincerely commiserate him and his brethren who are occupied in "keeping" in order the "partition wall" between the prairie "wolves" and the sheep. Should this occupation engross much of their time, however, the latter may rapidly succumb to rot developed within the fold.

When the attention of Dr. Storer was called to the above libel upon his professional character, he immediately wrote the note which follows, and which we transfer to our pages from a copy of the original one sent to Dr. Pitcher. It speaks for itself, and in our opinion is couched in terms which correctly, but very mildly, describe the "unwarrantable attack."

Boston, June 19th, 1856.

*Dear Sir:* In the last number of the "Peninsular Journal of Medicine," I find a scurrilous and most unwarrantable attack upon me, by one of its conductors, or with their permission.

As the President of the American Medical Association is senior editor of that Journal, an importance may be attached to the article in question, which would otherwise be unnoticed. I feel called upon, therefore, to state that the allegation, so far as it has reference to me, is *false*, and that, for more than thirty years, it has been my constant effort to maintain the honor of our profession—and no man has the right to call me "his friend" who would intimate otherwise.

Respectfully, David Humphreys Storer.

I will thank you to insert the above in your next number, that my reply may follow the slander. D. H. S.

In the July number of the Peninsular Journal, the editors have published this note, and they accompany it with certain remarks—before presenting which, we must call attention to the fact of the introduction of the word "malicious," in place of "scurrilous," as used by Professor Storer. The misquotation is rather worse than the misspelling and the misnaming—in fact, it is a *miss* altogether. That the word is substituted, is very apparent, not only because it is in the Peninsular's printed version, but also because Dr. Pitcher (or whoever wrote the article) has used the word "scurrility," placing it in quotation marks, as if from Dr. Storer's note. Here are the editorial comments to which we refer.

"*Matters Personal—Medical Card.*—Dr. H. Perdbeau, German Homœopathic Physician, has the honor to offer his services to the inhabitants of Washington and vicinity. Dr. P. has been during the last three years assistant of the celebrated Dr. Hoffendahl, in Boston, and feels confident to merit a share of the public patronage. Children and Female diseases, bowel and summer complaints, fall especially under his treatment.

"Office on D street, 2d door west of 9th, where he will be found from 9 to 12 in the morning, and 4 to 6 in the evening.

"Residence I street, 133, between 20th and 21st.

"References—Dr. Hoffendahl; Dr. Wesselhœft; Humphreys R. Storer, Professor of Midwifery in the M. M. College in Boston, &c."—*Tri-Weekly Intelligencer*, May 13, 1856.

In our issue for June, at pages 564 and 5, after alluding to the preceding card in language which we supposed might give offence to the author of it, the following paragraph occurs, which is now reproduced, in order that the reader of the note from Professor Storer may be fully aware of the nature of the "attack" made upon him through this Journal, and of the degree of "scurrility" which entered into the structure of the language employed in making an inquiry, which we supposed would naturally arise in the mind of every medical reader of the Intelligencer, and which we believed Dr. Storer would thank us for giving him an opportunity to answer in a manner much more emphatic and direct than he has chosen to do, in the note which he requests us to publish. Here is the paragraph referred to:

"This statement is made as a reason for instituting the inquiry, what relation this Dr. Storer bears to the medical profession of Boston? And whether it is possible that one of this name, affiliated to our friend H. R. Storer, M.D., can have so far forgotten the respect due his natural and professional allegiance, as to allow his name to be merged in the advertisement of a mountebank?"

Our readers, we think, will fail to perceive any "allegation" made by us, affecting the professional honor of Professor Storer, or, an "intimation" even, that he had done aught to tarnish that of the body to which he belongs, and by which he has been distinguished.

If David Humphreys Storer is not the person referred to by the Homœopathist, we have done him no wrong; but if he is the physician referred to, the wrong has been done by another party, who should be called upon to make the required statement, instead of ourselves. Feeling that we can acquit ourselves, both of the desire and the design, to wound either the honor or the sensibilities of a professional gentleman, we shall at all times hold ourselves under obligation, when convinced of either, to make the amplest reparation the case will admit of.

We confess ourselves at a loss to discover any more "emphatic and direct manner" of answering any charges than that of pronouncing them "*false*," as Prof. Storer has done; perhaps other modes may be known to Dr. H.—a description of them, and of the process of using them, would be interesting.

So far from "failing to perceive" any allegation affecting Prof. Storer's professional honor, in the first article from the "Peninsular," we do very distinctly see it, and condemn it. Let those who read, judge for themselves. If it were not intended, by the coarse article first published in reference to this matter, to give an "intimation, even," that Dr. Storer had done something to tarnish the honor of the body to which he belongs, "and by which he has been distinguished," why pen the mischievous sentences at all? If no harm has been done, why find fault? We fail to observe any logical accumen in this attempt of the editor to disengage himself from the awkward and self-assumed position of a false accuser. If he wished to discountenance the unlawful and unauthorized use of a professional brother's name, by one whom he terms "a mountebank," the proper way was open—could he not write a note, privately, to Dr. Storer, and ascertain the facts? Why should he jump at a conclusion proved to be so "lame and impotent?" What right has he publicly to assail a well-known and honorable physician, upon no other grounds than the seeing his name appended to an advertisement? Had he thus asked the question of Professor Storer, and ascertained that his discreditable suppositions were, *in any degree*, correct, it would then have been the time to rebuke—but *not until then.* An editor who can, at short intervals, distribute his opinions over the entire country, should never allow his private dislikes to influence his official language, nor empty the vials of his personal wrath upon his victims by means of his printed pages. We are informed that the most pleasant relations have hitherto existed between the parties involved in this disagreeable affair, and are therefore the more astonished at the whole performance. We are enabled, on the authority of Professor Storer himself, to present the *facts* in relation to his acquaintance with the Dr. Perabeau, whose very culpable conduct in using Dr. Storer's name without his permission, has caused so much trouble. A full and prompt apology is demanded from *him*, for thus originating the difficulty, no less than from the editor of the Peninsular Journal, for publishing and denouncing an innocent man, without any knowledge of the truth, so easily to be obtained from him.

Dr. Storer gives to us, in writing, the following information.

"During the last winter, a gentleman of the name of *Perabeau* attended the medical lectures in our school. He was always at my lectures, and was a faithful student. It occasionally happened, after my lecture, finding he was going toward the center of the city, I would offer him a seat in my vehicle, as I had often done to others, under similar circumstances. I have not seen, nor heard of him since—nor did I know he was now alive, until the appearance of the note from the editors, prefixed. It seems he is now a practising physician—homœopathist—and he has referred to *me*. In doing so, *he has done wrong*—because the inference may be drawn that *I had allowed him* to do so. He does not say this, however—feeling that he was a faithful pupil, he has seen fit to refer to me, as men are every day referring to others, for their character, without asking the right to do so—thus showing their confidence in their authority. As an homœopathist, for it seems he is one now, I know *nothing* of him—but as a medical pupil, I knew and respected him."

Had it been the fervent wish of the editors of the Peninsular Journal of Medicine to bring the Homœopathist Perabeau into very favorable notice, there could hardly have been a more effectual method adopted; for by thus obliging a well-known medical professor to testify to his faithfulness and zeal as a *medical pupil*, they give him an enviable position before the community, and those amongst whom he resides are all the more likely to employ him, as it is presumable his present zeal is commensurate, at least, with his former devotion to study. Never was a trumpet blown more to the discomfiture and disgrace of its owner—we only hope the result of the ill-advised blast may teach the wisdom of avoiding that extravagant tendency to *polemics* which disfigures some of our medical journals. We recommend to their attention the good adage, "*look before you leap;*" and the somewhat more modern one, "be sure you are right—then go a head!"

It can hardly be expected that practitioners, who (like Prof. Storer) are constantly and fully occupied, will hereafter think it worth their while—after having for thirty years, or more, striven for the honor of the profession, as well as to preserve their own—to cultivate, at great sacrifice of professional emolument, the personal acquaintance of their more distant brethren at meetings of the American Medical Association, if those who stand highest in the ranks of that body, either willfully or carelessly commit, or authorize, such outrageous attacks upon a professional brother through the medium of their pages.—*Boston Journal.*

